# How to best distribute written patient education materials among patients with rheumatoid arthritis: a randomized comparison of two strategies

**DOI:** 10.1186/s12913-018-3039-4

**Published:** 2018-03-27

**Authors:** Aniek A. O. M. Claassen, Cornelia H. M. van den Ende, Jorit J. L. Meesters, Sanne Pellegrom, Brigitte M. Kaarls-Ohms, Jacoba Vooijs, Gerardine E. M. P. Willemsen-de Mey, Thea P. M. Vliet Vlieland

**Affiliations:** 1 Department of Rheumatology, Sint Maartenskliniek, 6500 GM Nijmegen, The Netherlands; 2 Department of Rheumatology, Radboud University Medical Center, 6525 GA Nijmegen, The Netherlands; 3 Department of Rheumatology, Leiden University Medical Center, 2300 RC Leiden, The Netherlands; 4 Patient Research Partner, Leiden, The Netherlands; 5 Patient Research Partner, Katwijk, The Netherlands; 6 Patient Research Partner, Nijmegen, The Netherlands; 7 Department of Orthopedics, Leiden University Medical Center, 2300 RC Leiden, The Netherlands

**Keywords:** Patient education, Distribution strategy, Rheumatoid arthritis, Self-management, Dissemination

## Abstract

**Background:**

The aim of this randomized controlled trial was to evaluate the effect of a ‘supply on demand’-distribution strategy, compared to an ‘unsolicited supply’-distribution strategy, on the use of a care booklet and clinical outcomes among patients with rheumatoid arthritis (RA). In addition, differences in socio-demographic and clinical characteristics between users and non-users were explored.

**Methods:**

As part of regular care the care booklet was distributed among RA-patients of two hospitals in the Netherlands. 1000 patients received the care booklet by mail, whereas another 1000 received an information letter with the option to order the care booklet. Four months after distribution, a random sample of 810 patients (stratified by hospital and distribution method) received a questionnaire on the use of the booklet, social-demographic and clinical characteristics. To compare effects between the two distribution strategies and differences between users and non-users univariate and multilevel regression analyses were performed. Secondary analysis included a per-protocol analysis (excluding participants who did not order the care booklet).

**Results:**

One hundred ninety four patients in the ‘unsolicited supply’ and 176 patients in the ‘supply on demand’ group (46%) returned the questionnaire. In the ‘supply on demand’ group 106 (60.2%) participants ordered the care booklet. In total, no difference was found in use between the ‘unsolicited supply’-group (23.2%) and the ‘supply on demand’-group (21.6%) (OR 0.9 CI:0.6–1.5). However, the proportion of users among patients in the ‘supply on demand’-group who ordered the booklet (35%) was significantly higher than in the ‘unsolicited supply’-group (OR 1.9 CI:1.1–3.2). Regardless of distribution method, use of the care booklet was associated with being married (OR 2.4 CI:1.2–4.6), higher disease activity (mean difference 0.5 CI: 0.0–1.1), more activity limitations (mean difference 0.2 CI: 0.1–0.4), use of corticosteroids (OR 1.9 CI:1.0–3.5), perception of disease course as fluctuating (mean difference 1.4 CI:0.5–2.3) and higher educational needs (mean difference 9.7 CI: 2.9–16.6).

**Conclusions:**

From an economic and environmental perspective a ‘supply on demand’-distribution strategy could be recommended. Results of this study provide starting points to optimize further implementation strategies of a care-booklet in routine care.

**Trial registration:**

ISRCTN registry (ISRCTN22703067). Retrospectively registered 27 March 2017.

**Electronic supplementary material:**

The online version of this article (10.1186/s12913-018-3039-4) contains supplementary material, which is available to authorized users.

## Background

As a result of pain, fatigue and limitations in daily activities and participation, patients with rheumatoid arthritis (RA) have a significantly impaired health related quality of life [1]. Apart from the consequences of the disease, patients with RA deal with different medical treatments and a variety of healthcare providers during their course of illness. Therefore, supporting self-management is an important element of non-pharmacological care [2–4]. This encompasses activities, skills and interventions which allow patients to learn to cope with the consequences and treatment of their chronic illness and to take care of themselves [2, 3]. To enhance self-management, multiple interventions with similar content like face-to-face education and patient information booklets and leaflets, both on paper and online are available for patients with RA. Preferences may vary with regard to mode of delivery [5, 6].

One strategy to augment effective self-management is the use of patient care booklets. A care booklet can support patients with a chronic condition to play an active role in managing their disease and treatment by providing information and tools for monitoring symptoms, prepare consultations, record treatment targets and medication [4, 7, 8]. Furthermore, a care booklet can be used as prompt to enhance the communication between patient and healthcare provider during a consultation [7, 9]. Research on the effectiveness of different interactive care booklets for osteoarthritis, diabetes and back pain, shows improvement in illness perceptions and clinical outcomes [10–12]. In addition, as the goals of care booklets are to inform patients and to enhance their role in managing their disease, it could be expected that the use of a care booklet can have positive effects on a patients educational needs [4] and self-efficacy [13].

Despite positive effects and recommendations, the use of care booklets is in general suboptimal. Previous studies in diabetes care [12], hypertension [14] and mental health [15] suggest that the percentage of patients using a care booklet is variable (< 55%). Other studies suggest that a care booklet may be particularly useful for newly diagnosed patients [16] and that its content should preferably be tailored to the patient’s unique information needs and preferences, and perceptions about their disease, self-management and usefulness of the booklet [4]. However, little is known about optimal strategies to introduce a care booklet to patients [7] and data on head to head comparison of different distribution strategies on the use of educational material is not available. Usage after ‘unsolicited supply’ (i.e. sending a care-booklet without being requested) may differ from that after ‘supply on demand’ (i.e. offering the option to order a care booklet), as patients who are offered the option to order a care booklet might be better motivated to use it, ultimately resulting in better outcomes.

1. In order to study the effect of different distribution strategies on the use of a recently developed care booklet for patients with RA, the aims of the present study were to evaluate the effect of a ‘supply on demand’ distribution strategy and an ‘unsolicited supply’ distribution strategy for an RA care booklet regarding its usage, and patients’ educational needs, self-efficacy and illness perceptions to explore differences in patient and clinical characteristics between users and non-users of the care booklet.

## Methods

### Study design

In this multicentre randomized controlled trial two distribution strategies of a care booklet for patients with RA (‘unsolicited supply’ or ‘supply on demand’) were compared. The study was executed between September 2013 and May 2014 at the outpatient clinics for rheumatology of two hospitals in two regions of the Netherlands (Leiden University Medical Center (LUMC), Leiden and the Sint Maartenskliniek Hospital (SMK), Nijmegen). The Institutional Review Board of the University Medical Centre, Nijmegen (protocol number 2013/292) and the Medical Ethics Review Committee of the University Medical Centre, Leiden (protocol number: P13.202) both waived ethical approval, as the Medical Research Involving Human Subjects Act did not apply to this study.

### The RA care booklet

The initiative to develop a care booklet for patients with RA was taken by regional patient organizations and further developed as a collaborative project of RA patients, healthcare providers and researchers. The process of development and content of the interactive self-management “RA care booklet” (Zorgwijzer Reumatoïde Artritis©) is described in an additional file [see Additional file 1].

### Procedure

As part of regular care, the care booklet was distributed among patients with RA visiting the outpatient clinics of the departments of rheumatology of the LUMC and the SMK between September–December 2013. Because funding for printing booklets was restricted, for each outpatient clinic 1000 care booklets were available for distribution. Patients eligible to receive a care booklet were selected from the outpatient clinics’ registries by a data manager if they fulfilled the following criteria: 1) diagnosed with RA, 2) aged ≥18 years old and 3) having a future scheduled visit with a rheumatologist. Two distribution strategies were randomly applied by the researchers (AAOMC and SP) in each outpatient clinic concerned 1): ‘unsolicited supply’ of the care booklet free of charge to the home-address of patients accompanied by an introductory letter on behalf of the medical head of the department of rheumatology and 2) ‘supply on demand’: mailing an introductory letter about the care booklet on behalf of the medical head of the department of rheumatology to the home-address of patients with the option to order the care booklet free of charge. Patients could order the RA care booklet by sending back a reply card. Randomisation to the two distribution strategies in the outpatients of the LUMC was stratified for participation in an other ongoing study (yes/no), based on advise of the local review board of the LUMC.

Four months after distribution of the care booklet or the information letter about the care booklet, the subgroup of patients who were selected for the evaluation study received information about the study, a questionnaire, as well as a consent form. Reminders were sent after two weeks.

### Participants

**Fig. 1:**
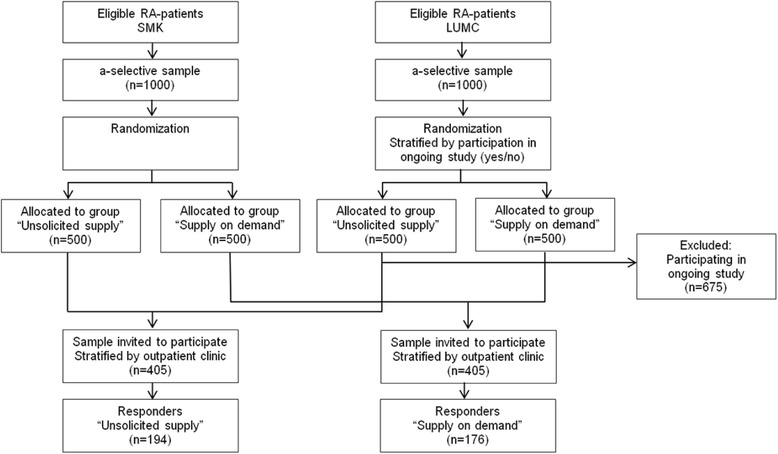
Participants selection from the Sint Maartenskliniek Hospital and Leiden University Medical Centre and response rate

For the current study we planned on inviting half of the patients who were randomized to the two distribution strategies for the evaluation (500 from each outpatient clinic). However, patients from the LUMC who were participating in another on-going study were excluded (remaining participants *n* = 310). A total sample of 810 patients (stratified by outpatient clinic) were randomly invited to participate in this study (Fig. 1).

### Assessments

The questionnaire comprised questions on the ordering and usage of the care booklet. In addition, a number of questions on demographic, clinical and psychosocial characteristics and educational needs were incorporated. The maximal time needed to complete the questionnaires was estimated to be 30–60 min.

#### Use of the care booklet

First patients were asked whether they had received the care booklet (in the ‘unsolicited supply’-group) or had received the care booklet after ordering it (‘supply on demand’-group).

Second, patients were asked whether they used the care booklet in the past 4 months. Answer-options included: “no”, “yes, I read (parts of) the care booklet”, “yes, I made notes in the care pass”, “yes, I discussed (parts of) the care booklet/pass with my healthcare provider”, “yes, I used the care booklet in a different way, namely….”. Multiple answers were possible. Patients who answered “no”, or stated that they did not receive or ordered the care booklet were classified as “non-users”. Patients answering 1 or more of the “yes”-answers were classified as “users”.

#### Demographic characteristics

Demographic characteristics included age, gender, ethnicity (native or foreign, based on land of birth, land of birth mother and father), marital status (based on being married, divorced, wido*w*/widower, never been married), education (≤12 years of education, > 12 years of education) and work status (based on having paid work or not (retired, unemployed, disabled, student, housewife/man) yes/no)).

#### Clinical characteristics

Included disease duration (years since diagnosis) and medication use (yes/no of *nonsteroidal anti*-*inflammatory drugs* (*NSAIDs*), corticosteroids, disease-modifying anti-rheumatic drugs (DMARDs), Biologicals, other).

The Rheumatoid Arthritis Disease Activity Index (RADAI) was used to measure disease activity [17]. The RADAI is a 5-item self-registered measure of disease activity, which include; “arthritis activity over the past 6 months”, “arthritis activity today”, “arthritis pain today”, “morning stiffness today” and “severity of pain per joint”. A total score can be calculated by dividing the sum of scores by 5. Total score ranges from 0 to10 (higher score = more disease activity).

#### Activities of daily living

Limitations in activities were assessed by the Health Assessment Questionnaire Disability Index (HAQ-DI) [18]. The HAQ-DI has 20-items. Daily activities are scored on a 4-point scale (0 “without difficulty” – 3 “unable to do”). Overall score can be computed as the sum of domain scores, divided by the number of domains answered. Total scores can range from 0 to 3 (0 = least difficulty, 3 = extreme difficulty).

#### Educational needs

Educational needs were assessed using the Dutch version of the Educational Needs Assessment Tool (D-ENAT) [19]. The D-ENAT consist of 39 items, grouped into seven domains: managing pain, movement, feelings, arthritis process, treatments, self-help measures and support systems. RA patients are asked to indicate how important it is for them to know more about certain topics. A total score can be calculated, ranging from 0 to 156 (higher score indicate higher educational needs).

#### Self-efficacy

The Dutch General Self-efficacy Scale (GSES) was used to measure self-efficacy [20]. The GSES has 10 items of which a total score can be calculated. Patients are asked about the belief that their own actions are responsible for successful outcomes, on a scale from 1 (not at all true) to 4 (exactly true). Higher scores on the GSES, ranging from 10 to 40 reflect higher self-efficacy.

#### Illness perceptions

To measure illness perceptions the Revised Illness Perception Questionnaire (IPQ-R) was used [21]. The IPQ-R has items divided into 7 subcategories, beliefs about: RA being acute or chronic (timeline; range 6–30), RA having a variety of consequences (consequences; range 4–20), RA having a fluctuating disease course (timeline cyclical; range 6–30), RA being under personal control (personal control; range 6–30), the ability to control one’s health due to treatment (treatment control; range 5–25), the level of understanding RA (illness coherence; range 5–25) and RA causing a variety of emotional states (emotional representation; range 6–30). High scores on the timeline, consequences, and cyclical dimensions represent strongly held beliefs about the number of symptoms attributed to the illness, the chronicity of the condition, the negative consequences of the illness, and the cyclical nature of the condition. High scores on the personal control, treatment control and coherence dimensions, represent positive beliefs about the controllability of the illness and a personal understanding of the condition.

### Statistical analysis

#### Sample size

To detect a 15% difference in proportions of users, assuming 40% use in the ‘unsolicited supply’-group versus 55% in the ‘supply on demand’-group, 372 participants (*n* = 186 for per group) would be needed (power 0.8, alpha 0.05) [22]. Estimations of proportions of use were based on user-rates in previous studies, which varied between 36 and 55% [12, 14, 15]. A 15% difference between groups was considered to be relevant. Considering the comprehensiveness of the questionnaire we assumed a 45% response rate [19, 23], yielding a sample size of 810 patients to be invited for this study.

Data were analysed using Stata version 13.0 (https://www.stata.com/). Descriptive statistics were provided as mean and standard deviation (SD) and numbers with percentages (%), where applicable. Imputed data were used for regression analyses. Missing data were imputed using Imputation by Chained Equations, which is an iterative multivariable regression technique, to obtain less biased results and preserve power [24].

In order to analyse the effect of distribution strategy (“unsolicited supply” / “supply on demand”) on the use of the care booklet a multilevel logistic regression analysis was performed using “outpatient clinic” as a random effect, using “use of care booklet” as dependent variable and distribution strategy as independent variable (intention-to-treat analysis).

Multilevel linear regression analyses were used to explore the effect of the distribution strategy on educational needs, self-efficacy and illness perceptions including “outpatient clinic” as a random effect.

Additionally analyses were repeated on a per-protocol basis including only those participants in the ‘supply on demand group’ who indicated that they had ordered the care booklet.

Finally, univariate logistic and linear regression analyses were used to explore the differences in age, gender, ethnicity, living status, level of education, work,disease duration, disease activity, activity limitations, use of medication, educational needs, self-efficacy and illness perceptions and between users and non-users of the care booklet. When needed analyses were corrected for “outpatient clinic” and “distribution strategy”.

A statistical significance level of *p* < 0.05 (two-sided) was adopted for all analyses.

## Results

### Patients’ demographics and characteristics

From the 810 invited patients, 194 patients in the ‘unsolicited supply’ group and 176 patients in the ‘supply on demand’ group (total 370 (45.7%)) provided written consent for participating in the present study and returned the questionnaire (Fig. 1). Table 1 shows the patient characteristics per distribution-group. No significant differences were found between the two groups.

**Table 1:** Demographics and characteristics of patients allocated to the two distribution strategies and as total group

	Group	Group	Total
	“unsolicited supply”	“supply on demand”	*n* = 370
	*N* = 194	*N* = 176	
Patient characteristics			
Gender (female), n (%)	137 (70.6)	114 (64.8)	251 (67.8)
Age (years), mean (SD)	65.0 (11.5)	65.3 (12.4)	65.4 (11.8)
Ethnicity (native), n (%)	175 (91.2)	157 (90.2)	332 (90.7)
Married, n (%)	132 (72.5)	122 (72.2)	254 (72.4)
Level of Education (> 12 years), n (%)	75 (40.1)	67 (39.0)	142 (39.6)
Work (paid), n (%)	46 (24.3)	37 (22.4)	83 (23.5)
Outpatient clinic (LUMC), n (%)	66 (34.0)	49 (27.8)	115 (31.1)
Clinical characteristics			
Disease duration (years), mean (SD)	17.5 (11.9)	16.4 (12.9)	17.0 (12.4)
Disease activity, RADAI (0–10), mean (SD)	2.5 (1.9)	2.8 (2.0)	2.6 (1.9)
Activity limitations HAQ-DI (0–3), mean (SD)	0.9 (0.7)	0.9 (0.7)	0.9 (0.7)
Medication, n (%)			
NSAIDs	66 (34.0)	62 (35.2)	128 (34.6)
Corticosteroid	33 (17.0)	27 (15.3)	60 (16.22)
DMARDs	131 (67.5)	123 (69.9)	254 (68.65)
Biologicals	82 (42.3)	87 (49.4)	169 (45.68)
Care booklet			
Ordered the care booklet, n (%)	–	106 (60.2)	–
Care booklet, n (%)			
Non-user	139 (76.8)	134 (78.4)	273 (77.6)
User	42 (23.2)	37 (21.6)	79 (22.4)

*RADAI* Rheumatoid Arthritis Disease Activity Index, *HAQ-DI* Health Assessment Questionnaire Disability Index

#### Distribution strategy

The ‘unsolicited supply’-group included 28 patients who did not recall to have received the care booklet, who were assigned as non-users. In the ‘supply on demand’ group 106 (60.2%) patients had ordered the booklet.

In the total study group, 79 patients (22.4%) used the care booklet, 42 (23.2%) in the ‘unsolicited supply’-group and 37 (21.6%) in the ‘supply on demand’ group (OR 0.9 CI: 0.6–1.5, (intention-to-treat analysis). Logistic regression analysis with “outpatient clinic” as a random effect, yielded similar results. In the total group, no differences were found between the two distribution strategies in any of the secondary outcomes (Table 2). Educational needs were slightly lower in the ‘supply on demand’-group, however this difference was not significant. A sensitivity analysis on complete cases yielded similar results.

**Table 2:** Differences in secondary outcomes between the two distribution strategies

	Group “unsolicited supply” N = 194	Group “supply on demand” N = 176	Difference (95% CI)
Educational needs, D-ENAT(0–156), mean (SD)	81.3 (26.9)	75.7 (27.5)	−4.2 (−9.9–1.5)
Self-efficacy, GSES, mean (SD)	32.4 (5.7)	32.6 (5.4)	0.2 (−1.0–1.3)
Illness perceptions, IPQ-R, mean (SD)			
Timeline (6–30)	24.7 (4.6)	24.4 (5.4)	−0.4 (−1.3–0.6)
Consequences (4–20)	18.9 (4.8)	18.8 (4.5)	− 0.1 (− 1.1–0.9)
Timeline cyclical (6–30)	13.9 (3.4)	14.2 (3.5)	0.3 (− 0.4–1.0)
Personal control (6–30)	19.0 (3.6)	19.7 (3.6)	0.7 (0.0–1.5)
Treatment control (5–25)	17.8 (2.9)	17.9 (3.0)	0.1 (− 0.5–0.7)
Illness coherence (5–25)	17.6 (3.8)	17.4 (3.6)	− 0.2 (− 1.0–0.6)
Emotional representation (6–30)	13.8 (4.3)	14.1 (4.1)	0.3 (− 0.6–1.1)

*D-ENAT* (Dutch) Educational Needs Assessment Tool, *GSES* General Self-efficacy Scale, *IPQ-R* Revised Illness Perception Questionnaire

A total of 37 (35%) of the patients who ordered the care booklet actually used the care booklet. In the per-protocol analysis a significant relation between distribution strategy and actual use of the care booklet (OR 1.9 CI: 1.1–3.2) in favour of the ‘supply on demand’-group was observed. Similar to the intention-to-treat analysis in the per-protocol analysis no differences were found in any of the secondary outcomes.

#### Comparison of users and non-users of the care booklet

Apart from self-ordering of a care booklet, a number of factors appeared to be associated with its eventual usage: the users were more often married (OR 2.4 CI: 1.2–4.6) than non-users. Users had a higher disease activity (Δ 0.5 CI: 0.0–1.1), more activity limitations (Δ 0.2 CI: 0.1–0.4), used corticosteroids (OR 1.9 CI: 1.0–3.5) more often, experienced the course of RA as fluctuating (Δ 1.4 CI: 0.5–2.3) and had higher educational needs (Δ 9.7 CI: 2.9–16.6) compared to non-users. No differences between users and non-users were found in other patient and clinical characteristics (Table 3).

**Table 3:** Differences in patient and clinical characteristics between users and non-users of the care booklet

	Non-users	Users	OR (95% CI)
	*N* = 273	*N* = 79	
Patient characteristics			
Gender (*female*), n (%)	182 (66.7)	58 (73.4)	1.4 (0.8–2.4)
Age *(years),* mean (SD)	65.3 (12.2)	64.7 (9.6)	Δ − 0.7 (−3.7–2.3)
Ethnicity (*foreign*), n (%)	27 (10.0)	6 (7.7)	0.8 (0.3–1.9)
Married, n (%)	181 (69.4)	64 (84.2)	2.4 (1.2–4.6)**
Level of education *(> 12 years*), n (%)	106 (39.9)	32 (41.6)	1.1 (0.6–1.8)
Clinical characteristics			
Disease duration *(years),* mean (SD)	17.3 (12.8)	15.9 (11.2)	Δ −1.5 (−4.6–1.7)
Disease activity, RADAI (0–10), mean (SD)	2.5 (1.9)	3.0 (2.0)	Δ 0.5 (0.0–1.1)**
Activity limitations HAQ-DI (0–3), mean (SD)	0.9 (0.7)	1.1 (0.7)	Δ 0.2 (0.1–0.4)**
Medication, n (%)			
NSAIDs	97 (35.5)	29 (36.7)	1.0 (0.6–1.7)
Corticosteroid	39 (14.3)	19 (24.1)	1.9 (1.0–3.5)**
DMARDs	185 (67.8)	58 (73.4)	1.3 (0.8–2.3)
Biologicals	123 (45.1)	42 (53.2)	1.4 (0.8–2.3)
Educational needs, D-ENAT(0–156), mean (SD)	75.3 (27.5)	86.0 (26.6)	Δ 9.7 (2.9–16.6)**
Self-efficacy, GSES, mean (SD)	32.3 (5.9)	33.1 (4.1)	Δ 0.8 (−0.6–2.2)
Illness perceptions, IPQ-R, mean (SD)			
Timeline (6–30)	24.5 (4.6)	25.1 (3.9)	Δ 0.6 (−0.5–1.8)
Consequences (4–20)	18.6 (4.7)	19.8 (4.5)	Δ 1.3 (−0.2–2.3)
Timeline cyclical (6–30)	13.6 (3.6)	15.1 (3.0)	Δ 1.4 (0.5–2.3)**
Personal control (6–30)	19.2 (3.7)	19.6 (3.5)	Δ 0.4 (−0.5–1.3)
Treatment control (5–25)	17.8 (3.0)	17.9 (2.7)	Δ 0.1 (−0.6–0.9)
Illness coherence (5–25)	17.4 (3.7)	18.0 (3.5)	Δ 0.5 (−0.5–1.4)
Emotional representation (6–30)	13.9 (4.3)	14.0 (3.6)	Δ 0.1 (−1.0–1.1)

*D-ENAT* (Dutch) Educational Needs Assessment Tool, *RADAI* Rheumatoid Arthritis Disease Activity Index, *HAQ-DI* Health Assessment Questionnaire Disability Index, *GSES* General Self-efficacy Scale, *IPQ-R* Revised Illness Perception Questionnaire

**Significant for *p*-value ≤0.05

## Discussion

This is the first study on the effect of distribution strategy on use of an interactive self-management care booklet for patients with RA. Overall 1 out of 5 patients (22%) used the care booklet. No differences were found in numbers of users of the care booklet between the two distribution methods (23.2% in the ‘unsolicited supply’-group, versus 21.6% in the ‘supply on demand’-group). Consequently, no differences between the distribution-groups were found on secondary clinical and patient related outcomes. However, the proportion of users was higher among patients who had ordered the booklet in the supply on demand group (35%) as compared to the unsolicited supply group.

When comparing users with non-users, we found significant differences in marital status, disease activity, activity limitations, use of corticosteroids, educational needs and illness perceptions (timeline cyclical).

We hypothesized that patients in the ‘supply on demand’-group would use the care booklet more often than patients in de ‘unsolicited supply’-group. As patients who took the step of ordering the care booklet, might be more eager to use it. Indeed, when only including patients who ordered the care booklet, the relative percentage of users in this distribution group rises from 21.6% to 35%. This difference is relevant from an economic perspective. Considering that the costs of the care booklet are about €1.50, it seems to be a better strategy to only send the care booklet to patients who order it. Overall this may lead to less expenses in the distribution of the booklet, as less money is lost to sending care booklets to patients who do not use them [see Additional file 1]. This is also an important point from an environmental perspective. Not only less costs are made when distributing the care booklet on demand, but also fewer care booklets are unnecessarily printed and distributed, making the ‘supply on demand strategy more sustainable. Further research on cost-effectiveness of the care booklet should be done to confirm these results.

In total, only 22% of the participating RA-patients reported to have used the care booklet. This is even lower than use of care booklets reported in previous studies [12, 14, 15]. Low usage of the care booklet in the group of patients who ordered the care booklet might be caused by high expectations patients had when requesting the care booklet. Cuperus et al. (2013) reported reported that patients’ perceptions about the usefulness of a care booklet has impact on the actual use of the booklet [4]. It is conceivable that patients perceived the care booklet to be useful when they ordered it, because it was provided by their outpatient clinic for free. But once received patients did not perceive the booklet to be a useful to manage their condition. However, it is debatable if this rate should be considered as low, given that 1 out of 5 RA patients uses a low-cost self-management tool, after a reasonably simple dissemination strategy. For instance, previous research in diabetes care reported a user-rate of 36%, six months after disseminating a booklet to diabetes patients. However, in this previous study dissemination was incorporated in other intervention activities, in which the booklet was introduced by health care providers in an educational meeting [12]. In a second study on this diabetes patient booklet, implementation strategies were even more thoroughly; relevant patient data were recorded in the booklet before handing it to the patient and patients were asked to bring the booklet to every clinic visit [7]. This led to a usage rate of 76%. These results suggest that to reach a higher uptake of care booklets a more enhanced dissemination strategy is needed and that the care booklet should be embedded in a larger intervention.

Different theories and methods have been developed to enhance and facilitate the adaptation or ‘uptake’ of new ideas or innovations like promoting self-management. In their description of the process of dissemination, Greenhalgh, et al. suggested that identification and use of appropriate communication and distribution channels is important [25]. To the best of our knowledge, the present study is the first to evaluate the effect of distribution strategies of a care booklet, on actual use among patients. A previous study on a cancer screening decision aid video did look at multiple strategies for distribution, including a supply on demand strategy [26]. They concluded that an automatic distribution strategy to all eligible patients is more effective than a strategy which relies on a patient’s initiative. However, they only evaluated the number of videos that were disseminated to eligible patients, not if the videos were actually used or watched. This could explain why their conclusion is not in line with our results, as we do not find an unsolicited supply strategy to be superior. Also, the screening decision aid was intended for patients to be seen before their doctor’s visit. The video was sent to all patients with an appointment and not necessarily to eligible patients. Screening for appropriate diagnosis before sending a care booklet or the option to order one seems therefore to be helpful in a distribution approach [7, 12, 26].

In a recent review on strategies for dissemination of recommendations and guidelines towards patients, Schipper et al. showed that many ‘opinion’-papers have recommendations about dissemination strategies. [27]. Only 1 out of 21 of the included studies in their review produced empirical evidence. In this respect, our study contributes to higher level of evidence for the effect of dissemination strategies on the uptake of patient information, and specific a care booklet. On the basis of the latter review, Schippers et al. described recommendations on how to involve patients in the development and dissemination process of guidelines to improve uptake [27]. The involvement of patients in health research is increasingly accepted and promoted, as a significant aspect of ensuring the development of high quality, relevant and necessary research [28]. In line with this development, a number of representatives from regional associations for patients with rheumatic diseases in The Netherlands were closely involved in the development of our care booklet. The active involvement of patients in the initiation and execution of the project assured that the perspective of patients was optimally taken into account resulting in a care booklet tailored to the preferences and perceptions of RA patients about the disease and options of self-management. The involvement of patients in the development of the care booklet may have led to the perceived usefulness of the booklet among users (mean rating 8 on a scale from 0 to 10).

We analysed differences between users and non-users of the care booklet, in order to explore if we could identify certain target groups based on patient and clinical factors. One difference that we found was that users were married more often, than non-users. It could be hypothesized that patients are motivated by their partner to use the care booklet. This is in line with previous research that RA-patients need social support to better manage their chronic condition and take an active role in their own care process [29]. Clinical outcomes showed that users had a higher disease activity, more activity limitations, used corticosteroids more often and experienced their RA as having a fluctuating disease course more often compared to non-users. We could hypothesize that these outcomes reflect more disease severity and that, thus, disease severity is associated with use of booklet. We also found that users of the care booklet had higher educational needs. Based on our study design, it is not possible to conclude whether these outcomes are a target group characteristic or changed because of use of the care booklet. However, these results do offer starting points for further research into identifying target groups when distributing care booklets.

This study has some limitations that need to be mentioned. First, although generalizability increases by including patients from two different outpatient clinics, there was a risk for selection bias. The LUMC had considerably fewer eligible patients (diagnosed RA) to be approached for the dissemination of the care booklet. Additionally, patients from the LUMC who were participating in another on-going study were not allowed to participate in the present study. This should be taken into consideration when interpreting the results. Second, our choice not to include a baseline assessment could be argued. As a result we were not able to analyse the effect of the care booklet over time. However, the dissemination of the care booklets prior to inviting patients to participate in the study prevented socially desirable use as part of a research project.

## Conclusions

In conclusion, this randomized controlled trial shows that distribution strategy (unsolicited or supply on demand) does not influence the absolute number of RA patients eventually using a care booklet. Therefore, no influence of distribution strategy on clinical outcomes were found. The proportion of patients using the care booklet was somewhat higher in those who had ordered it on demand as compared to unsolicited supply. From an economic and environmental perspective a ‘supply on demand’ distribution strategy seems to be superior compared to a ‘unsolicited supply’ strategy. Our findings provide starting points to optimize further implementation strategies of a care-booklet by targeting specific subgroups of patients or by integrating the care booklet in the routine care.

## Additional file

Additional file 1: The RA care booklet - Information about the development and content of the RA Care booklet and an estimation of costs for each distribution strategy. (DOCX 21 kb)

## Abbreviations

D-ENAT: Dutch Educational Needs Assessment Tool
*DMARDs*: *D*isease-modifying anti-rheumatic drugs
GSES: General Self-efficacy Scale (GSES)
HAQ-DI: Health Assessment Questionnaire Disability Index
IPQ-R: Revised Illness Perception Questionnaire
LUMC: Leiden University Medical Center
NSAIDS: N*onsteroidal anti*-*inflammatory drugs*
RA: Rheumatoid arthritis
RADAI: Rheumatoid Arthritis Disease Activity Index
SMK: Sint Maartenskliniek Hospital
