# Supplementary material for: How to best distribute written patient education materials among patients with rheumatoid arthritis: a randomized comparison of two strategies
Source: BMC Health Serv Res. 2018 Mar 27;18:211. doi: 10.1186/s12913-018-3039-4 (PMC5870684; doi:10.1186/s12913-018-3039-4)
Supplement: Supplementary file 1 — The RA care booklet - Information about the development and content of the RA Care booklet and an estimation of costs for each distribution strategy. (DOCX 21 kb) [file 12913_2018_3039_MOESM1_ESM.docx]

**Additional file 1 - The RA care booklet**

A number of representatives from regional associations for patients with rheumatic diseases in The Netherlands expressed their need for a self-management tool. Therefore, the “RA care booklet” (Zorgwijzer Reumatoïde Artritis^©^) was developed as a collaborative project of healthcare providers and rheumatoid arthritis (RA) patients. We used a stepwise approach based on the developmental process proposed by Francis et al. (2008), which included: determining objectives and functionalities within a project team of researchers and patients, professional photography, graphic design and text editing, composing draft versions and consultation with stakeholders about the initial set up and draft versions [1]. For the consultation 10 patients, 4 rheumatologists and 5 clinical nurse specialists were asked in multiple rounds for written feedback.

The care booklet comprises 60 pages of information about RA and its treatment, living with RA and self-management of RA. The care booklet also incorporates a separate hand-out, the "RA care pass" (Zorgpas RA, 26 pages), allowing patients to record personal information like medical history, use of medication and monitor symptoms like pain, fatigue and disease activity, using the DAS28-score. Additionally, points to consider for upcoming consultations with health care providers and goal setting can be recorded in the care pass.

In the present study the care booklet was distributed using two strategies. Table s1 shows a estimation of material costs per 100 approached patients following these two distribution strategies.

Table s1 Estimated costs when approaching 100 patients following the two distribution strategies.

|  | | Unsolicited supply | Supply on demand |
| --- | --- | --- | --- |
| Care Booklet + pass printing costs | | € 1.41 | €1.41 |
| Postage and package costs | |  |  |
|  | Care booklet | € 3.12 | € 3.12 |
|  | Informational letter | - | € 0.78 |
|  | Reply card | - | € 0.78 |
| Total costs per patient that receives care booklet | | € 4.53 | €6.09 |
| Costs invested per 100 approached patients | | €453.00 ^a^ | €396.20 ^b^ |

^a^ € 4.53 x 100 = €453.00

^b^ €6.09 x 60 (number of people that order the care booklet based on present study) + € 0.78 x 40 (number of people who do not order the care booklet) = €396.20

**Reference List**

1. Francis N, Wood F, Simpson S, Hood K, Butler CC. Developing an 'interactive' booklet on respiratory tract infections in children for use in primary care consultations. Patient Educ Couns 2008, 73: 286-293.
